# Malpractice awareness among surgeons at a teaching hospital in Pakistan

**DOI:** 10.1186/1754-9493-6-26

**Published:** 2012-11-06

**Authors:** Asfandyar Sheikh, Sajid Ali, Sadaf Ejaz, Marium Farooqi, Syed Salman Ahmed, Imran Jawaid

**Affiliations:** 1 Dow Medical College, Dow University of Health Sciences, Karachi, Pakistan

**Keywords:** Malpractice, Negligence, Knowledge, Attitudes, Practices, Teaching hospital, Pakistan

## Abstract

**Background:**

The duty of a doctor to take care presumes the person who offers medical advice and treatment to unequivocally possess the skills and knowledge to do so. However, a sense of responsibility cannot be guaranteed in the absence of accountability, which in turn requires a comprehensive medical law system to be in place. Such a system is almost non-existent in Pakistan. Keeping the above in mind, we designed this study to assess the knowledge, attitudes and practices of surgeons regarding malpractice at a tertiary care center in Pakistan.

**Methods:**

This was an observational, cross-sectional, questionnaire-based study conducted during a three month period from 31st March, 2012 to 30th June, 2012 at Civil Hospital, Karachi. Surgeons who were available during the period of our study and had been working in the hospital for at least 6 months were included. Self-administered questionnaires were distributed after seeking informed, written consent. The specialties included were general surgery, cardiothoracic surgery, neurosurgery, ophthalmology, otolaryngology, plastic surgery, pediatric surgery, orthopedic surgery, oral and maxillofacial surgery and gynecology and obstetrics. The study questionnaire comprised of four sections. The first section was concerned with the demographics of the surgeons. The second section analyzed the knowledge of the respondents regarding professional negligence and malpractice. The third section assessed the attitudes surgeons with regard to malpractice. The last section dealt with the general and specific practices and experiences of surgeons regarding malpractice.

**Results:**

Of the 319 surgeons interviewed, 68.7% were oblivious of the complete definition of malpractice. Leaving foreign objects inside the patient (79.6%) was the most commonly agreed upon form of malpractice, whereas failure to break news in entirety (43.9%) was most frequently disagreed. In the event of a medical error, majority (67.7%) were ready to disclose their error to the patient. The most common perceived reason for not disclosing the error was threat of a claim or assault (90.9%). Majority (68.3%) believed that malpractice had a negative effect on reputation. Only 13(4.1%) had received at least one legal claim for damages. Only about three-fourths (75.5%) had the habit of frequently obtaining informed consent from the patients. 83(26.0%) expressed reluctance in accepting a case that was deemed to be difficult. Financial gains and liabilities were responsible for biased approach in 8.5% and 12.2% of the respondents respectively.

**Conclusion:**

There is a dire need of programs aimed at increasing awareness among practicing surgeons in our setup. Proactive measures are required for the formulation of an efficient system of litigation. Physician accountability will not only arouse a greater sense of responsibility in them, but will also augment the confidence placed by patients on the healthcare system.

## Background

Recent advances in medical technology and protocols have seen a proportional increase in the expectations of both the patients and the physicians. Medicine has become a prosperous business, partly attributable to the escalating healthcare costs that are prevalent in most setups. However, this commercialization of medicine has redefined the role of physicians as lifesavers. This implies that doctors no longer pay the amount of time and attention that is required and expected from them. The aptitude of a physician is no longer judged by his proficiency in handling difficult cases, but by his ability to handle the colossal amount of patient load that is imposed on him by the hospital authorities.

The duty of a doctor to take care presumes the person who offers medical advice and treatment to unequivocally possess the skills and knowledge to do so. The great Winston Churchill rightly said “The price of greatness is responsibility”. However, a sense of responsibility cannot be guaranteed in the absence of accountability, which in turn requires a comprehensive medical law system to be in place. The formulation of such a regimen is dependent upon the consistent and effective application of the rule of law to all aspects of civil life, including the healthcare system. However, such a regimen is almost non-existent in Pakistan, where instances of medical negligence are neither documented nor redressed. The only cases that come into limelight are those related to celebrities, such as those of Huma Wasim and Fauzia Wahab to name a few [1,2].

The legal system of the United States covers malpractice under the tort laws. By definition, a tort is an act (not necessary illegal) that causes harm to a person. Negligence is just one of the different types of torts, the others being intentional torts and quasi-torts. The person who suffers a tortious injury is entitled to receive compensation for damages. These damages can range from minor cosmetic defects to permanent disabilities with long term implications or even death. In developed countries, the concept of professional liability insurance has been implemented to cover these damages. However, for a negligence case to be established, the plaintiff must prove four elements, namely duty, breach of duty, damage or injury and breach of duty being a proximate cause of the damage [3].

European tort laws are distinct from those in the United States in two different ways. Firstly, in most European legal systems, non-economic damages are decided by judges guided by detailed legal rules, whereas in the United States, the jury, directed by its conscience, is responsible for making such decisions [4]. The second difference relates to the presence of a thriving “tort industry” in the United States, which has led to both the lawyers and general public taking advantage, with mass litigation campaigns amounting to millions of dollars [4]. Such an industry is non-existent in Europe, owing to meager damage amounts decided by the judges and lower contingency fees compared to the United States [4].

The Pakistan Penal Code defines personal injuries under article 332 titled “Hurts” [5]. According to the code, there are five kinds of injuries: itlaf-i-udw (dismemberment of any limb or organ), itlaf-i-salahiyyat-i-udw (impairment of the functioning, power or capacity of an organ, or permanent disfigurement), shajjah (injury on the head or face of any person, which does not amount to itlaf-i-udw or itlaf-i-salahiyyat-i-udw), jurh (which leaves a mark of the wound) and miscellaneous hurts [5]. All of the above may be punishable by qisas (blood money) based on the principle of “an eye for an eye,” monetary compensation and/or imprisonment [5].

The Law and Justice Commission of Pakistan instituted malpractice laws under “The Allopathic System (Prevention of Misuse) Ordinance” [6]. The Pakistan Medical and Dental Council (PMDC), established under an Ordinance in 1962, is a statutory, autonomous regulatory body in Pakistan that deals with the registration of medical practitioners, and is responsible for maintaining the highest standards of medical practice. If a complaint is received for professional misconduct, the disciplinary committee under PMDC is responsible for taking disciplinary action. However, malpractice litigation is a phenomenon unheard of in the Pakistani society, which may arise from a lack of trust on the legal system prevalent in the country.

Doctors are amongst the most sued professionals in the world. This finding is consistent with the fact that about 45,000 and 98,000 fatalities that occur each year in the United States may be attributed to malpractice [7]. Similarly, the National Health Service Litigation Authority dealt with 8,655 claims of clinical negligence and 4,346 of non-clinical negligence in 2011 [8]. An increased risk of malpractice claims is particularly found in surgical specialties. Researchers estimate that as many as one half to two thirds of inpatient adverse events result from inappropriate surgical care [9-11]. These events result from a variety of factors such as including inexperienced surgeons, excessive workload with resultant fatigue, unavailability of required technology, poor supervision and lack of proper communication [12-16].

In this study, the investigators aimed to assess the knowledge, attitudes and practices of surgeons at a tertiary care center in Pakistan. This study is unique in the sense that no reports have been previously published from a country that needs serious reformation of malpractice laws.

## Materials and methods

### Study setting

This was an observational, cross-sectional, questionnaire-based study conducted during a three month period from 31st March, 2012 to 30th June, 2012 at Civil Hospital, Karachi, which is a public sector, tertiary care hospital. It is the second largest hospital in Metropolitan Karachi, and provides free healthcare to patients, majority of whom belong to low socio-economic class. The hospital employs more than 1400 doctors in different specialties and capacities. Surgeons form a considerable proportion of this number, which can largely be attributed to the fact that surgery is one of the most popular career choices among medical students in Karachi, as reported by Rehman and Huda [17,18].

### Study participants

**Figure 1:**
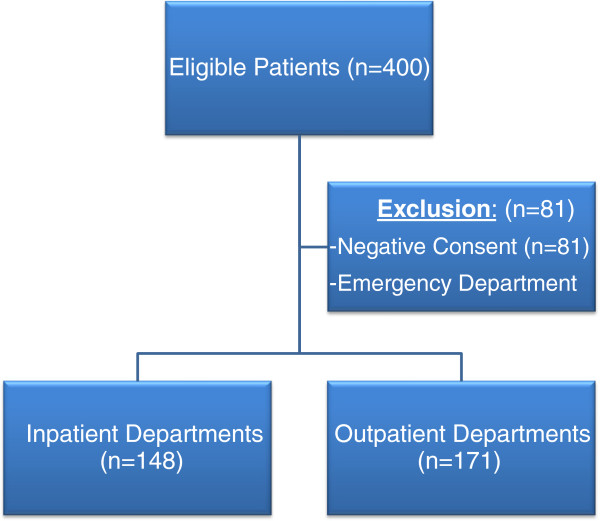
Recruitment summary.

We attempted to interview all surgeons who were available during the period of our study. Thus, convenience sampling was employed. The names of surgeons working in a specific ward were obtained from the central database of the corresponding ward. They were then approached and self-administered questionnaires were distributed after seeking informed, written consent. For those who were busy, questionnaires were handed over to be filled in their spare time, and collected the day after. Figure 1 provides a graphical representation of the recruitment protocol.

#### Inclusion and exclusion criteria

The study included all the surgeons who were working in the hospital for at least 6 months. They were recruited from both the inpatient and outpatient departments. The specialties included were general surgery, cardiothoracic surgery, neurosurgery, ophthalmology, otolaryngology, plastic surgery, pediatric surgery, orthopedic surgery, oral and maxillofacial surgery and gynecology and obstetrics. Surgeons working in the emergency department were not included, due to the lack of comparability with other specialties. Those who gave consent in negative were also excluded. Table 1 provides a representation of the departments included.

**Table 1:** Distribution of the respondents according to departments

	**Inpatient N(%)**	**Outpatient N(%)**
General Surgery	33(10.3%)	42(13.2%)
Cardiac Surgery	12(3.8%)	6(1.9%)
Neurosurgery	10(3.1%)	12(3.8%)
Ophthalmology	14(4.4%)	17(5.3%)
Otolaryngology	13(4.1%)	18(5.6%)
Plastic Surgery	9(2.8%)	11(3.4%)
Pediatric Surgery	13(4.1%)	12(3.8%)
Orthopedic Surgery	7(2.2%)	9(2.8%)
Oral and Maxillofacial Surgery	15(4.7%)	19(6.0%)
Gynecology and Obstetrics	22(6.9%)	25 (7.8%)
Total	148(46.4%)	171(53.6%)

### Ethical review

The Ethical Review Board of Dow University of Health Sciences approved the study. The respondents were informed of their right to refuse at any time of the study. Confidentiality and anonymity of the data was maintained at all times. The protocol was designed according to the guidelines laid down by the Helsinki Declaration [19].

### Operational definitions

#### Negligence

Failure to exercise the care that a reasonably prudent person would exercise in like circumstances [20].

#### Malpractice

A type of negligence that includes injuries caused by a physician’s “neglect or unskillful management” in violation of the trust placed in that practitioner [21].

#### Adverse outcome

An injury sustained by a patient in the course of receiving medical treatment, which was not a reasonably foreseeable side effect of the treatment.

#### Medical error

A human error that occurs when a healthcare provider chooses an inappropriate method of care, or in case of the right choice, executes it incorrectly [22].

#### Misdiagnosis

A medical error that involves incorrect or faulty diagnosis of a disease.

#### Informed consent

Consent given in the presence of three components: disclosure, capacity and voluntariness [23]. It involves full disclosure of the necessary information along with adequate apprehension by the patient. Presence of external influence or pressure nullifies the consent.

#### Remuneration

Damages or reimbursements paid by the surgeons as a compensation of negligence.

#### Aggressive medicine

Utilizing every medication, technology and procedure possible in order to treat a condition.

#### Defensive medicine

Providing supportive or palliative care when medical futility has been established [24].

### Study protocol and questionnaire

The study questionnaire was designed with the help of the Departments of Surgery and Community Medicine, Dow University of Health Sciences. Thorough review of the literature was undertaken in order to design the best possible questionnaire. A pretest was done on an initial sample of 10 surgeons and the questionnaire was edited accordingly.

Before administration of the questionnaire, the respondents were asked general questions about malpractice, and their answers were compared with legal definitions and graded accordingly. The questionnaire comprised of four sections. The first section, which was concerned with the demographics of the surgeons, consisted of age, gender, marital status, comorbidities, years of experience and medical specialty. Assessment of workload was done by inquiring the number of hours of daily work and sleep. Those working in multiple centers (including clinics) were also required to select the appropriate options in the questionnaire. Defects or impairments in general health (eg farsightedness), that could hamper surgical performance, were also noted. Some of these variables were derived from the studies of Meadow, Taragin and Abbott et al. [25-27].

The second section analyzed the knowledge of the respondents regarding professional negligence and malpractice. A series of 20 different forms of malpractice were listed in a tabulated manner. Surgeons’ opinion on whether each of these forms came under the heading of malpractice was scored on a Likert scale of 1–5, with 1 denoting strong disagreement for a reason, 2 denoting a lesser degree of disagreement, 3 denoting neutrality, 4 denoting a lesser degree of agreement and 5 expressing strong affirmation for a reason. The third section assessed the attitudes surgeons with regard to malpractice. A list of yes/no questions appraising their attitudes were included. The last section dealt with the general and specific practices and experiences of surgeons regarding malpractice. Again a series of yes/no questions were included in order to assess their practices.

### Analysis of data

Our data did not have any missing values, as special emphasis was placed on the completion of the questionnaire at the time of administration. Data from the questionnaire was entered in SPSS (Statistical Package for the Social Sciences) version 17 for analysis and the results were compared. Descriptive statistics formed the mainstay of the statistical analysis. P values were calculated to determine the significance of association between variables and were based on the Chi-square test. A P value of less than 0.05 was considered to be significant.

## Results

### Demographics

A total of 319 out of 400 questionnaires were returned giving a response rate of 79.8%. The age of the respondents ranged from 23 years to 48 years, with a mean age of 26.2±8.1 years. 176(55.2%) respondents were female, whereas 143(44.8%) were male, giving a male to female ratio of 1:1.2 in our sample. Majority [166(52.0%)] was single, followed by those who were married [136(42.6%)], widowed [13(4.1%)] and divorced [4(1.3%)].

**Figure 2:**
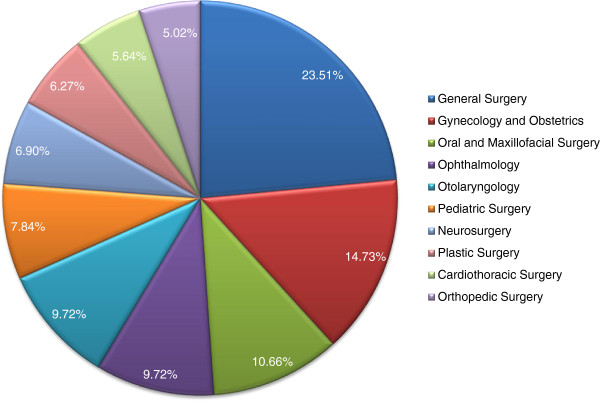
Specialty distribution of the respondents.

179(56.1%) respondents were house-officers, 113(35.4%) were undergoing post graduate training, while 27(8.4%) had already completed their post graduate training. About one-fourth [78(24.5%)] were part of the teaching staff. Mean monthly salary was Rs46,510±9,358 (~USD500 ± 100). General Surgery [75(23.5%)] was the most common subspecialty, followed by Gynecology and Obstetrics [47(14.7%)], Oral and Maxillofacial Surgery [34(10.7%)], Ophthalmology [31(9.7%)], Otolaryngology [31(9.7%)], Pediatric Surgery [25(7.8%)], Neurosurgery [22(6.9%)], Plastic Surgery [20(6.3%)], Cardiothoracic Surgery [18(5.6%)] and Orthopedic Surgery [16(5.0%)]. Figure 2 provides a graphical representation of this data. Majority [192(60.2%)] of the respondents also worked in other centers, with clinics [92(30.7%)] being the most common workplace, followed by other hospitals [73(22.9%)] and centers not related to medicine [21(6.6%)].

The most common chronic condition that the respondents suffered from was hypertension [63(19.7%)], followed by diabetes [19(6.0%)], and cardiovascular diseases [11(3.4%)]. A considerable proportion [40(12.5%)] was visually impaired, whereas [9(2.8%)] and [5(1.6%)] had defective hearing and speech respectively. The mean number of hours spent working daily (7.8±2.5 hours) was more than the mean number of hours spent sleeping (6.5±1.4 hours).

### Malpractice

A striking 68.7% of the respondents were oblivious of the complete definition of malpractice as mentioned above. The percentage was higher in lower education groups (82.1% in house-officers, 59.3% in those undergoing post graduate training and 18.5% in those who had already completed their training; P<0.05). Only 9(2.8%) of the respondents were aware of the four elements of a malpractice case, namely duty, breach of duty, damage or injury and breach of duty being a proximate cause of the damage [3].

### Knowledge

**Figure 3:**
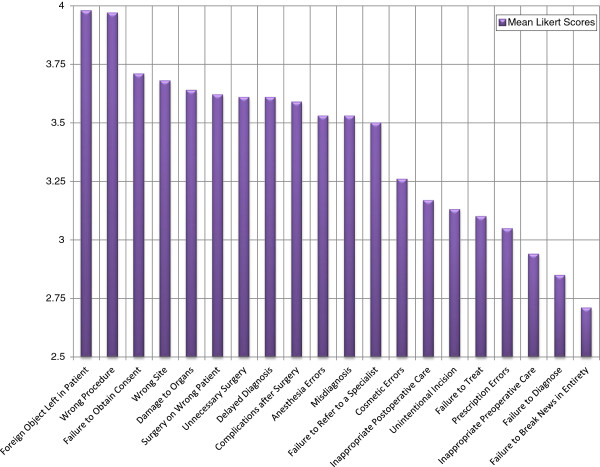
Mean likert scores for different forms of malpractice.

**Figure 4:**
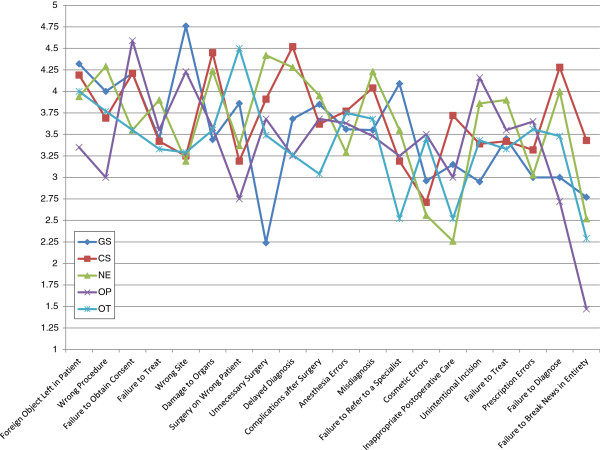
Changes in mean likert scores for different forms of malpractice across 5 specialties (Part 1 of 2).

**Figure 5:**
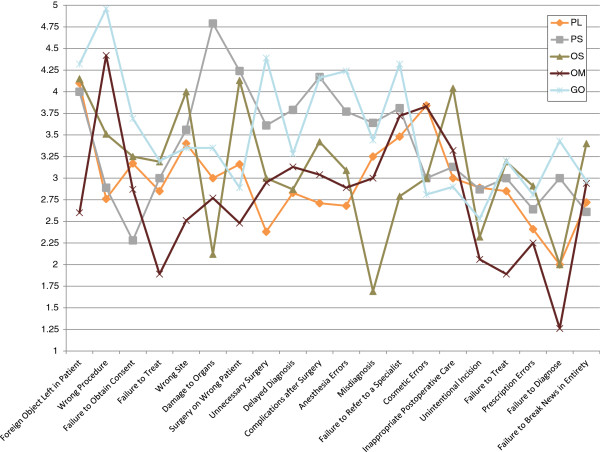
Changes in mean likert scores for different forms of malpractice across 5 specialties (Part 2 of 2).

Table 2 provides a summary of the mean Likert scores of the opinions of the respondents for each form of malpractice, with Figure 3 providing a graphical representation. Table 3 gives a breakdown of these scores according to specialty with Figures 4 and 5 providing a line chart for the same. Leaving foreign objects inside the patient (79.6%) was the most commonly agreed upon form of malpractice, followed by performing the wrong procedure (70.2%), failure to refer (67.1%), wrong patient (66.8%), unnecessary surgery (65.5%), misdiagnosis (64.3%), surgery at wrong site (63.9%), damaging organs during surgery (62.3%), delayed diagnosis (56.2%), performing procedure without consent (56.1%), anesthesia errors (54.6%), complications after surgery (51.4%), unintentional incision (45.5%), failure to treat (43.6%), cosmetic errors (40.4%), inappropriate postoperative care (38.9%), prescription errors (36.1%), inappropriate preoperative care (29.8%), failure to diagnose (27.9%) and failure to break news in entirety (13.8%).

**Table 2:** Opinions of respondents on different forms of malpractice

	**Likert scores**	**Should there be damage claims?**	
	**Mean**	**Yes**	**No**
Foreign Object Left in Patient	3.98 ± 0.99	232(72.7%)	87(27.3%)
Wrong Procedure	3.97 ± 1.34	154(48.3%)	165(51.7%)
Failure to Obtain Consent	3.71 ± 1.19	135(42.3%)	184(57.7%)
Wrong Site	3.68 ± 1.06	173(54.2%)	146(45.8%)
Damage to Organs	3.64 ± 1.26	217(68.0%)	102(32.0%)
Surgery on Wrong Patient	3.62 ± 1.30	187(58.6%)	132(41.4%)
Unnecessary Surgery	3.61 ± 1.37	128(40.1%)	191(59.9%)
Delayed Diagnosis	3.61 ± 1.01	160(50.2%)	159(49.8%)
Complications after Surgery	3.59 ± 1.10	193(60.5%)	126(39.5%)
Anesthesia Errors	3.53 ± 1.05	197(61.8%)	122(38.2%)
Misdiagnosis	3.53 ± 1.09	95(29.8%)	224(70.2%)
Failure to Refer to a Specialist	3.50 ± 1.02	63(19.7%)	256(80.3%)
Cosmetic Errors	3.26 ± 1.24	136(42.6%)	183(57.4%)
Inappropriate Postoperative Care	3.17 ± 1.17	106(33.2%)	213(66.8%)
Unintentional Incision	3.13 ± 1.14	47(14.7%)	272(85.3%)
Failure to Treat	3.10 ± 1.06	71(22.3%)	248(77.7%)
Prescription Errors	3.05 ± 1.24	25(7.8%)	294(92.2%)
Inappropriate Preoperative Care	2.94 ± 1.14	33(10.3%)	286(89.7%)
Failure to Diagnose	2.85 ± 1.14	66(20.7%)	253(79.3%)
Failure to Break News in Entirety	2.71 ± 1.09	39(12.2%)	280(87.8%)

**Table 3:** Mean likert scores of opinions of respondents belonging to different specialties

	**GS**^**1**^	**CS**^**2**^	**NE**^**3**^	**OP**^**4**^	**OT**^**5**^	**PL**^**6**^	**PS**^**7**^	**OS**^**8**^	**OM**^**9**^	**GO**^**10**^	P-value*^+^
Foreign Object Left in Patient	4.32	4.19	3.94	3.35	4.00	4.10	4.00	4.15	2.60	4.32	NS
Wrong Procedure	4.00	3.69	4.29	3.00	3.77	2.76	2.89	3.51	4.42	4.96	NS
Failure to Obtain Consent	4.21	4.21	3.55	4.59	3.55	3.17	2.28	3.25	2.87	3.69	NS
Failure to Treat	3.45	3.42	3.90	3.55	3.33	2.85	3.00	3.19	1.89	3.20	NS
Wrong Site	4.76	3.25	3.19	4.23	3.29	3.40	3.56	4.00	2.51	3.35	NS
Damage to Organs	3.44	4.45	4.24	3.58	3.55	3.00	4.79	2.12	2.77	3.35	NS
Surgery on Wrong Patient	3.86	3.19	3.37	2.75	4.50	3.16	4.24	4.13	2.48	2.89	NS
Unnecessary Surgery	2.24	3.91	4.42	3.68	3.49	2.38	3.61	3.00	2.95	4.39	**<0.001**
Delayed Diagnosis	3.68	4.52	4.28	3.25	3.26	2.83	3.79	2.87	3.13	3.28	NS
Complications after Surgery	3.85	3.62	3.95	3.68	3.04	2.71	4.17	3.42	3.04	4.16	**0.002**
Anesthesia Errors	3.56	3.77	3.29	3.63	3.75	2.68	3.77	3.09	2.89	4.24	NS
Misdiagnosis	3.55	4.04	4.23	3.48	3.68	3.25	3.64	1.69	3.00	3.44	NS
Failure to Refer to a Specialist	4.09	3.19	3.55	3.25	2.52	3.48	3.81	2.79	3.72	4.32	**0.044**
Cosmetic Errors	2.96	2.71	2.56	3.50	3.45	3.84	3.00	3.00	3.83	2.81	**0.023**
Inappropriate Postoperative Care	3.15	3.72	2.26	3.00	2.52	3.00	3.13	4.04	3.32	2.90	**0.014**
Unintentional Incision	2.95	3.39	3.86	4.16	3.43	2.89	2.87	2.32	2.06	2.53	NS
Failure to Treat	3.45	3.42	3.90	3.55	3.33	2.85	3.00	3.19	1.89	3.20	NS
Prescription Errors	3.00	3.32	3.03	3.65	3.56	2.41	2.64	2.91	2.25	2.81	NS
Failure to Diagnose	3.00	4.28	4.00	2.72	3.48	2.00	3.00	2.00	1.26	3.43	NS
Failure to Break News in Entirety	2.77	3.43	2.52	1.47	2.29	2.72	2.61	3.40	2.94	2.97	NS

*Based on Chi-square Test ^+^NS = Not Significant.

^1^GS = General Surgery.

^2^CS = Cardiac Surgery.

^3^NE = Neurosurgery.

^4^OP = Ophthalmology.

^5^OT = Otolaryngology.

^6^PL = Plastic Surgery.

^7^PS = Pediatric Surgery.

^8^OS = Orthopedic Surgery.

^9^OM = Oral and Maxillofacial Surgery.

^10^GO = Gynecology and Obstetrics.

Failure to break news in entirety (43.9%) was the most commonly disagreed with form of malpractice, followed by failure to diagnose (39.1%), inappropriate preoperative care (35.8%), unintentional incision (34.1%), prescription errors (33.9%), unnecessary surgery (31.3%), inappropriate postoperative care (29.7%), cosmetic errors (26.6%), damaging organs during surgery (23.5%), failure to treat (22.9%), misdiagnosis (20.4%), performing the wrong procedure (19.4%), wrong patient (18.8%), failure to refer (17.9%), anesthesia errors (17.2%), performing procedure without consent (15.7%), complications after surgery (15.4%), delayed diagnosis (12.5%), surgery at wrong site (11.0%) and leaving foreign objects inside the patient (7.8%).

### Attitudes

Table 4 provides a summary of the attitudes and opinions of the respondents regarding malpractice. Majority (67.7%) of the respondents replied in affirmative when asked, “Will you disclose your error to the patient, if you get to know that your patient has been a target of malpractice?” However, different results were obtained for specific scenarios. The most common perceived reason for not disclosing the error was threat of physical or verbal assault (90.9%), followed by reputation being at stake (77.1%), patient not interested in knowing about the mistake (71.5%), patient not being able to understand the nature of error (55.2%), error being trivial (52.7%) and lack of strong ties between the patient and the surgeon (33.9%). A significant number of respondents was ready to correct their errors free of charge [280(87.8%)]. However, only a few were willing to offer monetary remuneration in lieu of their error [118(37.0%)]. The mean amount that the respondents were ready to pay was quite modest (Rs84,635±13,613[~USD900±150]). Majority [218(68.3%)] of the respondents believed that malpractice had a negative effect on reputation. Out of these, 129(59.2%) were of the view that the damage was serious but short term, 66(30.3%) believed that the damage was quite insignificant, while 23(10.5%) answered that damage was serious and long term. A striking 83.4% of the respondents reported that a considerable proportion of the patients seen in clinics could be counseled over the phone.

**Table 4:** Attitudes of respondents regarding malpractice

		
	Yes	No
Will you disclose your error to the patient, if you get to know that your patient has been a target of malpractice?	216(67.7%)	103(32.3%)
Will you disclose your error to the patient, if the error is trivial?	151(47.3%)	168(52.7%)
Will you disclose your error to the patient, if you feel that he won’t be able to understand what you are saying?	143(44.8%)	176(55.2%)
Will you disclose your error to the patient, if there is a threat of assault?	29(9.1%)	290(90.9%)
Will you disclose your error to the patient, if the patient is not interested in knowing about it?	91(28.5%)	228(71.5%)
Will you disclose your error to the patient, if you find that doing so would place your reputation at stake?	73(22.9%)	246(77.1%)
Will you disclose near misses?	97(30.4%)	222(69.6%)
Will you disclose your error to the patient, if you don’t know him very well?	211(66.1%)	108(33.9%)
Will you offer to correct your error, free of charge?	280(87.8%)	39(12.2%)
Will you offer monetary remuneration, if the error is not corrigible?	118(37.0%)	201(63.0%)
Will you continue to practice if you find that your senses are weakening, with the resulting negative impact on your career?	16(5.0%)	303(95.0%)
Are you willing to apologize for your mistake, if need be?	314(98.4%)	5(1.6%)
Do you think malpractice laws need reformation in Pakistan?	62(19.4%)	257(80.6%)

### Practices and experiences

Table 5 provides a summary of the practices and experiences of the respondents with regard to malpractice. 117(36.7%) respondents reported making at least one serious medical error in their careers. However, only 13(4.1%) had received at least one legal claim for damages.

**Table 5:** Practices and experiences of respondents with regard to malpractice

		
	Yes	No
Have you been through a serious medical error?	117(36.7%)	202(63.3%)
Did you disclose the error to the patient?	63(53.8%)	54(46.2%)
Did you feel relaxed after disclosing to the patient?	56(88.9%)	7(11.1%)
Have you ever received malpractice claims?	13(4.1%)	306(95.9%)
Have you ever been physically/verbally assaulted by the patient or their attendants?	147(46.1%)	172(53.9%)
Do you have a habit of frequently taking proper informed consent?	241(75.5%)	78(24.5%)
Do you keep yourself updated on innovations in medicinal protocols by a regular review of literature?	262(82.1%)	57(17.9%)
Do you avoid taking a case if you think it is difficult?	83(26.0%)	236(74.0%)
Are you more aggressive if there are financial gains associated with a case?	27(8.5%)	292(91.5%)
Are you more defensive if there are financial liabilities associated with a case?	39(12.2%)	280(87.8%)
Do you have a habit of frequently referring cases to specialists?	284(89.0%)	35(11.0%)
Do you order more tests than needed, in order to save yourself from liability/accountability?	131(41.1%)	188(58.9%)
Do you think that you provide adequate care to your patients?	301(94.4%)	18(5.6%)

A greater proportion of the surgeons in our sample reported practicing defensive (52.7%) rather than aggressive medicine (26.6%). About three-fourths (75.5%) had the habit of frequently obtaining informed consent from the patients. Only 262(82.1%) reported regular review of literature to keep themselves updated on the innovations in medicinal protocols. 83(26.0%) expressed reluctance in accepting a case that was deemed to be difficult. Financial gains and liabilities were responsible for biased approach in 8.5% and 12.2% of the respondents respectively. 5.6% of the respondents believed that they provided inadequate care to the patients.

## Discussion

Although a number of studies aimed at assessing the attitudes and practices of physicians regarding malpractice have been published, this is the first article of its kind that attempts to evaluate these parameters from a country that needs serious amendments in the relationship between its legal and medical systems. As such, the recommendations from this report can be used in the formulation of official guidelines by the concerned authorities.

The results of our study present a dismal picture of the surgeons’ acquaintance with the concept of negligence. Only a handful of those interviewed were able to answer general questions regarding malpractice correctly. This lack of knowledge comes partly from a paucity of adequate emphasis on professional and ethical behavior in our undergraduate medical curriculum. However, the major factor responsible for this is the fact that most physicians often practice freely and do not feel legally threatened by the current system of litigation. As such, endeavors are not undertaken to strengthen one’s comprehension of the topic, with the results being reflected in the attitude and practices of the respondents.

Although a significant proportion of respondents was ready to correct their errors free of charge, only a few were willing to offer monetary remuneration. This was also reflected in Table 3, which shows that most of the respondents were not in favor of damage claims being in place. This gives a slight hint of a strong disapproval from the physician community in case of an introduction of a stricter system of litigation in the future.

The doctrine of informed consent was not always followed in our sample. Complete disclosure was also found to be deficient. However, the individual scenarios seem to suggest the reasons behind the lack of abidance: Threat of assault was the most common reason of failure to disclose error. It must be noted here that physicians are human beings too, and are prone to the same fears that other humans. They must be assured of the basic right of security, as recent attacks on them have led to their increased migration to other countries [28].

Majority of the surgeons interviewed reported practicing defensive rather than aggressive medicine. This is quite surprising, given the precarious nature of the system of accountability in our setup, which should favor aggressive approach. A sense of greed was also found in some of the respondents as shown by a considerable proportion adopting secondary occupations and by the biased approach for financial gains. However, it is worthwhile to note here that the salaries of doctors in Pakistan are far from being adequate. A house-officer receives Rs24,000/month (~USD250), while a postgraduate receives Rs42,000/month (~USD450). This amount is often not in line with the expenses, especially in case of married physicians. Thus, they are forced to adopt secondary employments, and in doing so often neglect their duties in the hospital. Again, aggressive measures are needed from concerned authorities in order to rectify this problem.

### Limitations

The most important limitation for our study was that it was conducted in just one institute. Although, the hospital consists of a heterogeneous population coming from different backgrounds, it cannot predict the overall situation in the country. This notion is further supported by the fact that house officers formed the majority of the study respondents which may have biased the result in two ways. Firstly, house officers lack in practical experience and their opinions may not represent those of the entire physician population of the country. However, most senior professors refused to participate in the study citing lack of time and lack of potential implications of the study as reasons for non-participation (This is shown by the fact that only 2 of the house officers approached refused to give consent). Secondly, since house officers have only recently graduated, their gender distribution reflects that of the medical schools currently operating in the country, where females form a considerable majority. A recent article in DAWN reported a meager 88 males out of a total of 4839 applicants appearing for entry tests in medical schools in Sindh [29]. However, all efforts were made to ensure that neither of the study groups was under-represented.

The present deficiency of a proper litigation system in our setup also limits the usefulness of our results. Convenient sampling was employed, which is not truly representative of the population under study. However, since this was just an observational study, the sampling method did seem to fulfill its purpose.

## Conclusion

The general outcome of our study was quite disappointing. Although moderate to high scores were obtained for some forms of negligence, the overall awareness regarding malpractice remained relatively low in our sample. The attitude of the respondents was also discouraging, as majority was not ready to be held accountable for their wrongdoings. Similarly, the practices also demonstrated a deficiency of the appropriate level of care that is expected from physicians in general, and surgeons in particular. There is a dire need of programs aimed at increasing awareness among practicing surgeons in our setup. Proactive measures are required for the formulation of an efficient system of litigation. Physician accountability will not only arouse a greater sense of responsibility in them, but will also augment the confidence placed by patients on the healthcare system, which is a rare entity in our setup.

### Recommendations

In light of findings of our study, we would like to propose several measures to improve the prevailing lack of awareness regarding malpractice at our center and other centers nationwide.

• Special education campaigns which are conducted with the aims of promotion of awareness about professional negligence and its consequences should be arranged. These campaigns should be specially directed towards individuals belonging to the lower educational levels (e.g. those still in medical schools)

• Special emphasis should be placed on teaching medical law and ethics as separate subjects at the college level. A new syllabus should be defined for medical jurisprudence, which is often neglected as a subject in our setup. During residency, thorough literature review of malpractice suits should be considered mandatory, and hospital cases with possible legal implications should be discussed.

• There should be regular assessment of the clinical aptitude of physicians, in a manner comparable to that of developed countries such as the United States.

• The legal system of the country also needs reformation. The formulation of an impartial system of litigation should be granted utmost priority. A special committee, headed by the Chief Justice of Pakistan, should be formed that should look into all cases of negligence. Those found to be responsible should be brought to justice. However, such a change must come only after the shortcomings in the education system are overcome.

• An efficient warning system should also be developed, and physicians who are regularly found to be negligent should be stripped from their right to continue practice. Compensations should also be granted where possible. Once such a system is established, wide-scale advertisement campaigns aimed at arousing awareness amongst the general public should be setup.

• The rights of physicians must also be upheld. Stability should be brought to the structure of the insurance system in the country, and proper protocols for damage claims coverage should be introduced.

• The physical and verbal abuse exercised by certain people after an adverse event should be put to stop with provision of proper security by the hospital.

• The salaries of the physicians should be increased, and they should be entitled to fringe benefits and contingent rewards, in order to ensure a loyal and dedicated workforce.

## Abbreviations

PMDC: Pakistan medical and dental council; NHSLA: National health service litigation authority.

## Competing interests

The authors declare that they have no conflicts of interests.

## Authors’ contributions

AS conceived the topic of the study and was involved in designing the study and analyzing data. AS, SA, SE, MF, SS and IJ were involved in data collection. AS was involved in drafting the initial manuscript. SA, SE, MF, SS and IJ critically revised the manuscript, and their names are listed in decreasing order of their contributions. All authors have read and approved the final manuscript.
